# The Role of Probiotics in the Eradication of Helicobacter pylori and Overall Impact on Management of Peptic Ulcer: A Study Involving Patients Undergoing Triple Therapy in Bangladesh

**DOI:** 10.7759/cureus.56283

**Published:** 2024-03-16

**Authors:** Taslima Zaman, Ahsanul Haq, Rahnuma Ahmad, Susmita Sinha, Kona Chowdhury, Sultana Parvin, Mostofa Imran, Zaman U Humayra, Santosh Kumar, Mainul Haque

**Affiliations:** 1 Department of Gastroenterology, United Hospital Ltd, Dhaka, BGD; 2 Department of Biostatistics, RNA Biotech Limited, Dhaka, BGD; 3 Department of Physiology, Medical College for Women & Hospital, Dhaka, BGD; 4 Department of Physiology, Khulna City Medical College and Hospital, Khulna, BGD; 5 Department of Pediatrics, Gonoshasthaya Samaj Vittik Medical College, Dhaka, BGD; 6 Department of Medical Gastroenterology, Sheikh Russel National Gastroliver Institute & Hospital, Dhaka, BGD; 7 Department of Gastroenterology, Ibn Sina Medical College & Hospital, Dhaka, BGD; 8 Department of Plastic and Reconstructive Surgery, Ship International Hospital, Dhaka, BGD; 9 Department of Periodontology and Implantology, Karnavati School of Dentistry, Karnavati University, Gandhinagar, IND; 10 Karnavati Scientific Research Center (KSRC), Karnavati School of Dentistry, Karnavati University, Gandhinagar, IND; 11 Unit of Pharmacology and Therapeutics, National Defence University of Malaysia, Kuala Lumpur, MYS

**Keywords:** h. pylori treatment, helicobacter pylori, triple therapy, bangladesh, pud, peptic ulcer disease, probiotic microflora, probiotic bacterium

## Abstract

Background

*Helicobacter pylori* infection has been identified to cause constantly recurring inflammation, leading to gastrointestinal tract disorders, including carcinoma. The standard triple therapy (STT), used to eradicate *H.* *pylori,* includes two antimicrobials and a proton pump inhibitor for two weeks. Other drug regimens have also been developed since *H. pylori* exhibits antimicrobial resistance. These regimens, including probiotics, have been shown to lower adverse drug reactions (ADR), improve drug adherence, exert bacteriostatic effect, and reduce inflammation.

Objective

This study intended to explore probiotic intervention for improving eradication rates and mitigating adverse effects while administrating STT.

Methods

This prospective study was conducted from May to December, 2021, in the Department of Gastroenterology of Ship International Hospital, Dhaka, Bangladesh, to observe the effects of probiotics inclusion along with STT on *H.* *pylori* eradication. A total of 100 patients aged ≥18 years who tested positive for *H.* *pylori* were included. The experimental group (n=50) was given STT and probiotics, and the control group (n=50) was given only STT without probiotics for 14 days. Necessary follow-up was done six weeks after treatment. An independent sample t-test, chi-square test, and multiple regression analysis were used for statistical analysis.

Result

The odds of getting rapid urease test (RUT) negative results from positive were 2.06 times higher (95%CI= 0.95, 3.22, p=0.054) in the experimental group. ADRs were crucially towering in the control group (p=0.045) compared to the probiotics group. The probiotics group had a lower risk of having adverse effects by 0.54 times (95%CI=0.19, 0.84, p=0.032) than the control group.

Conclusion

Using probiotics and STT together to eradicate *H. pylori* may lower ADR and improve treatment adherence. It may also help terminate *H.* *pylori* infection more effectively. More research is required as *H. pylori *is very contagious and can ultimately cause life-threatening gastric cancer.

## Introduction

Peptic ulcer disease (PUD) is described as hydrochloric acid-provoked damage of the epithelial lining of the gastrointestinal tract, arising predominantly in the stomach and duodenum called gastric ulcer (GU) and duodenal ulcer (DU), respectively [1,2]. Australia-born Professor (Dr.) Barry James Marshall and Dr. John Robin Warren first reported in 1982 that the principal cause of PUD is infective disorders caused by curved gram-negative bacillus *Helicobacter pylori*, and later, in 2005, both obtained the Nobel Prize jointly for this discovery [3-5]. Earlier, it was believed that regular ingestion of alcohol and tobacco, psychological multiple disorders, nonsteroidal anti-inflammatory drugs (NSAIDs), and lifestyle issues were principal causes of PUD [6-8]. Marshall and Warren refuted the age-old concept of these causes of PUD [4,5,9].

It is known that *H. pylori* causes around 90-95% and 80-85% of DU and GU, respectively [4,10,11]. Multiple studies reported that more than 50% of the globe's populace is infected with *H. afpylori *[12,13], with a considerable difference in the frequency among countries and the prevalence observed within a country [12-15]. Nevertheless, all patients infected with *H. pylori *do not acquire PUD; only 10% of individuals develop PUD [10,16,17]. *H. pylori* is responsible for the chronic infection that triggers a chronic inflammatory process in the superficial epithelial layer of the stomach; later, it causes several gastrointestinal disorders, including carcinoma [18-21]. 

Hooi et al. steered a systematic review and meta-analysis to make public that the African (70.1%; 95%CI, 62.6-77.7) and Australian (Oceania) (24.4%; 95%CI, 18.5-30.4) continents had the maximum and minimum pooled generality of *H. pylori* infection, respectively. The pervasiveness rate of *H. pylori *infection varies in different nations with the lowest in Switzerland (18.9%) and the highest in Nigeria (87.7%) [15]. Congedi et al. published a scoping review that reported a decreased tendency of *H. pylori* infection among Australian citizens. Nevertheless, the scoping review could not confirm the infectious potential of *H. pylori* among susceptible clusters [22]. Another systematic review conducted by Peleteiro et al. reported that the highest and lowest rates of *H pylori* infection were found in Mexico (90%) and Finland (13.1%), respectively [23]. Although distinct differences are observed regarding *H. pylori* infection rates across the world, the general preponderance of *H. pylori* infection is around 50% of the worldwide populace [24,25].

The mortality rate because of *H. pylori* infection was considerably higher among the non-pharmacologically intervened group than in treated clusters (p<0.001) [26]. It has been ascertained that the deaths among pharmacologically intervened and non-intervened clusters were 4.1-5.9% and 5.5-7.6%, respectively [26,27]. *H. pylori *has been identified as the foremost jeopardizing factor in gastric carcinoma [28]. Stomach cancer is considered the fifth leading malignancy and stands in third position for carcinoma-related mortality around the globe [29]. It has been reported that 770,000 [30] to 800,000 [31] people passed away because of stomach cancer, and around 1.1 million fresh cases were seen in 2020 [30]. The rate of frequency of stomach carcinoma is influenced by sex. *H. pylori* and related diseases affect male subjects two-fold more than female counterparts [30]. It has been appraised that stomach cancer alone accounts for 7.7% of all carcinoma-related deaths [31]. It is thus strongly advocated to eradicate *H. pylori*, especially in seropositive individuals with cytotoxin-associated gene A (CagA) [32-36]. CagA is an external cancer-stimulating protein for humans generated by some strains of *H. pylori* [34,37].

Australian Dr. Thomas J. Borody introduced the bismuth-based triple therapy containing bismuth and two antimicrobials. This was the first successful effort to manage *H. pylori* abolition [38,39]. Goh et al. put forward the theory that the therapeutic intervention for *H. pylori *infection should include two antimicrobials (clarithromycin and amoxicillin) and a proton pump inhibitor (PPI) for two weeks [40]. This combination is often called standard triple therapy (STT). Additionally, in those cases of *H. pylori* infection that are isensitive to penicillin (amoxicillin), it is replaced with metronidazole [40,41]. Other than the mentioned antimicrobials, tetracyclines and fluoroquinolones are commonly used for *H. pylori* eradication [42-44].

The National Institutes of Health brought the first consensus report regarding eradicating *H. pylori* infection in 1994 [45]. Subsequently, the European *H. pylori *Study Group endorsed STT as the leading therapeutic strategy during the first Maastricht conference in 1997 [46]. Furthermore, multiple consensus guidelines published in the last two decades or more for the therapeutic intervention of *H. pylori* infection, such as Maastricht VI/Florence [47], the Toronto [48], Kyoto Global [49], Hong Kong recommendation [50], Taipei Global [51] and many more [52-56].

*H. pylori* is a gram-negative, spiral-shaped, microaerophilic, and highly infective pathogenic microbe; mounting antimicrobial resistance around the globe creates an alarming human health threat [57,58]. Many treatment regimes and attempts at eradicating *H. pylori* were unsuccessful because of resistance [42,59-61]. *H. pylori *attained 100-1000 times more resistance to multiple antimicrobials when *H. pylori* were grown in the matching floating form or cholesterol [62-65]. ﻿Another study revealed that *H. pylori*'s resistance to clarithromycin was 22.2%. Amoxicillin and metronidazole resistance in *H. pylori *were 1.2% and 69.2%, respectively. The resistance patterns in the United States and Europe were similar and resistance to metronidazole was found to be the highest (50-79%) and the least resistance was to amoxicillin (equal to or lower than 5%) [66]. Global resistance pattern against commonly prescribed antimicrobials for *H. pylori* infection eradication is high and ranges from 15-50% [67]. Multiple studies reported that among eight South Asian countries (Bhutan, Bangladesh, India, Indonesia, South Korea, Nepal, Sri Lanka, and Thailand), the antimicrobial resistance of *H. pylori* patterns is much higher (98%). There is a high incidence of self-purchasing of antimicrobials from community pharmacies, poor antimicrobial stewardship programs, and rapid alteration of geopolitical scenery and urbanization [68-71]. One global systematic review and meta-analysis also reported that 15% of *H. pylori *possesses either primary or secondary resistance towards clarithromycin, metronidazole, and levofloxacin in practically all world regions [72]. Nahar et al. reported from Bangladesh that *H. pylori* isolates were resistant to amoxicillin, clarithromycin, tetracycline, and metronidazole, and the resistance was determined to be 6.6%, 10%, 15%, and 77.5%, respectively [73]. Over a decade after the study by Mahar et al. [72], Aftab et al. reported that *H. pylori *isolates in Bangladesh showed resistance to metronidazole, levofloxacin, and clarithromycin, and resistance rates were 94.6%, 66.1%, and 39.3%, respectively [74].

Our planet faces treatment difficulties regarding infectious diseases because of antimicrobial resistance to almost all available antimicrobials, which poses an enormous global health threat [75,76]. Overall, antimicrobial resistance around the globe is the foremost public health issue, which equally affects *H. pylori* and related diseases because of the evolution of drug resistance. Moreover, *H. pylori* has been notified as the principal factor for gastric carcinoma with fatal consequences [77]. In this antagonist situation, researchers developed multiple other regimens, such as bismuth-containing quadruple (hybrid therapy (HT) including a PPI, bismuth, metronidazole, and tetracycline), sequential (﻿PPI plus amoxicillin followed by PPI, clarithromycin, and an imidazole), PCN (PPI, clarithromycin and nitroimidazoles), and concurrent or accompaniment (non-bismuth quadruple including PPI, clarithromycin, amoxicillin, and metronidazole) therapy to combat antimicrobial-resistant *H. pylori* infection beside SST [78-83]. However, even with all these efforts, *H. pylori* eradication often failed because of resistance to multiple antimicrobials [84], such as amoxicillin, clarithromycin, metronidazole, and levofloxacin [41,85].

﻿Diarrhea, constipation, nausea, vomiting, epigastric pain, flatulence, metallic taste, and pain in the abdomen, especially in the epigastric region, are frequently occurring adverse drug reactions (ADRs) following SST therapy to combat *H. pylori* infection [86,87]. Another study revealed that over 26% of the research participants had experienced ADRs [88]. Among them, 85% had gastrointestinal issues, such as gastrointestinal distress, nausea, uncomfortable or infrequent bowel movements (typically less than three times per week), loose motions, stomach upset, an eating disorder frequently accompanied by unrestrained body weight loss, and headache [88,89]. Another study revealed that over 45% of participants encountered principally gastrointestinal ADRs [90]. Again, around 5% of patients were unable to continue therapeutic intentions because of profound ADRs, and approximately 3% of cases were unable to take less than 80% of medication because of ADRs. Total or partial discontinuation or medication adherence to *H. pylori* therapeutic intervention frequently leads to eradication failure and promotes antimicrobial resistance [91-93]. Consequently, research analyses narrated that the foremost cause of *H. pylori* abolition failure is necessitous treatment incompliance that is related to STT-induced ADRs of prescribed drugs [88,94,95]. 

One metanalysis comprising 34 randomized control trials and over 9000 cases showed that the addition of *Bifidobacterium-Lactobacillus-Saccharomyces *and *Bifidobacterium-Lactobacillus-based* probiotics with diverse regimens of *H. pylori* extinction program resulted in minimizing ADRs and promoting medication adherence [96]. Probiotics competitively restrain the growth of *H. pylori* in the stomach with their bacteriostatic effect. Additionally, probiotics improve gastrointestinal microbiome status [97]. It has been reported that *Saccharomyces boulardii*, *Bacillus licheniformis*, *Lactobacillus acidophilus*, *Bifidobacterium *triple viable bacteria, and *Bacillus subtilis* dual viable bacteria are currently in clinical use in the management of *H. pylori* eradication program. These probiotics increase medicine adherence, cut back ADRs, especially antimicrobial-persuaded, mitigate the stomach mucosal chronic inflammation instigated by *H. pylori,* and increase the abolition rate of *H. pylori* when administered with various regimes as an adjuvant [97-107].

Problem statements of this study

Addressing the following problem statements can contribute significantly to developing more effective and patient-friendly *H. pylori *eradication therapies, ultimately improving treatment outcomes and reducing the global burden of *H. pylori*-associated diseases.

Efficacy Challenges in STT

One significant problem to address is the suboptimal extinction rate of *H. pylori* with the STT, consisting of a PPI, amoxicillin, and clarithromycin/levofloxacin. Despite being a widely employed treatment, the efficacy is compromised due to antimicrobial resistance and other factors [108].

Antibiotic-Related ADR

Another critical issue is the high incidence of ADRs associated with antibiotics, particularly clarithromycin and levofloxacin. Gastrointestinal disturbances, allergic reactions, and the development of antibiotic resistance are among the adverse effects that impact patient tolerance and compliance during *H. pylori* eradication therapy [109].

Impact of Dysbiosis on Treatment Outcome

The STT often disrupts the gut microbiota balance, leading to dysbiosis. This disturbance in the natural microbial community may contribute to prolonged recovery, increased susceptibility to infections, and other complications, necessitating exploring interventions to mitigate dysbiosis [110].

Need for Improved Treatment Strategies

Given the global rise in antibiotic resistance and the limitations of the STT, there is an urgent need to explore adjunctive therapies that can enhance eradication rates. Probiotics represent a promising avenue, but their specific role and mechanisms in improving treatment outcomes remain understudied and require comprehensive investigation [111].

Patient Non-Adherence and Treatment Failure

Poor patient compliance poses a significant challenge in *H. pylori* eradication therapy. Understanding the impact of probiotics on patient adherence and exploring strategies to improve compliance is crucial for achieving better treatment outcomes and reducing the risk of antibiotic resistance [94].

Variability in Probiotic Strains and Formulations

The variability in probiotic strains and formulations available in the market raises questions about their consistent effectiveness. Addressing the optimal selection, dosage, and duration of probiotic supplementation is essential to establish evidence-based recommendations for integration into *H. pylori* eradication regimens [112,113].

Objectives of the study

This study explores probiotic intervention for improving eradication rates and mitigating side effects in STT. It assesses the impact of probiotic supplementation on the eradication rate of *H. pylori* infection when combined with STT and evaluates the reduction in adverse effects of antibiotics of triple therapy, such as nausea, diarrhea, abdominal discomfort, metallic taste, headache, and joint pain and in determining the potential influence of probiotics on patient compliance and adherence to the prescribed treatment regimen. It also provides evidence-based recommendations regarding incorporating probiotics into STT for *H. pylori* extinction in clinical situations.

## Materials and methods

This was a prospective study conducted at the Department of Gastroenterology, Ship International Hospital, Dhaka, Bangladesh, from May to December, 2021. The study obtained ethical approval from the Institutional Review Board of Ship International Hospital (Formerly, Japan East West Medical College Hospital), Dhaka, Bangladesh (approval number: JEWMCH/IEC/01, dated March 5, 2021). Furthermore, written informed consent was obtained from all participants and the study adhered to the World Medical Association's Declaration of Helsinki, ethical principles for medical research involving human subjects. Additionally, research objectives and future publication plans were explained in detail to patients and guardians.

Inclusion and exclusion criteria

Inclusion Criteria

The following inclusion criteria defined the patients eligible for the study and provided a clear framework for selecting study participants: 1. Patients who were candidates for diagnostic endoscopy of the upper gastrointestinal tract (UGIT) with rapid urease test (RUT); 2. Patients who tested conclusively for *H. pylori* infection by RUT during the endoscopy; 3. Patients aged 18 years or older.

Exclusion Criteria

The following exclusion criteria ensured that the study population was well-defined and that the results were not confounded by factors that could affect the interpretation of the study outcomes: 1. Patients with a history of taking NSAIDs; 2. Patients with a previous history or report of hepatic, renal, or neoplastic diseases; 3. Pregnant women or lactating mothers; 4. Patients who received antibiotics or probiotics within four weeks before the study enrollment; 5. Patients with a known sensitivity or allergy to any drugs used in this study; 6. Patients who were unwilling to participate voluntarily in the study.

Sample size

**Figure 1:**
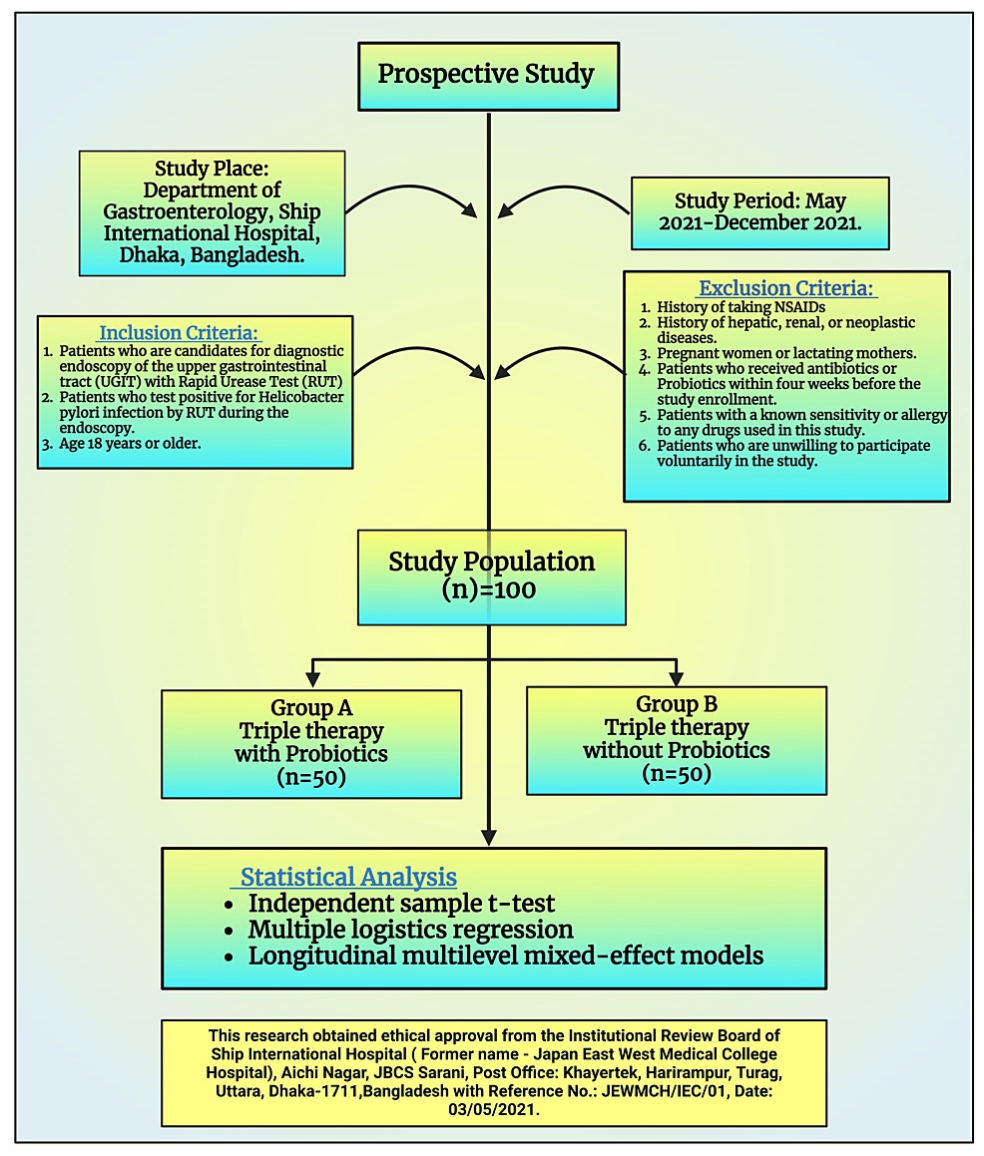
Schematic diagram showing the methodology of this study. Image credit: Sismita Sinha; with the premium version of BioRender [114] with the license number HM26K5BNK0

A simple random probability sampling technique was practiced to allocate the patients to the investigational and control groups. The sample size was calculated at a 5% level of significance and a confidence interval of 95%. The sample size was calculated by the following formula: N= Z2pq/ e2. Using the formula, sample size was n=(1.96)2x (0.5) x (0.5)/ (0.05)2 = 384. However, our sample size was kept at 100 due to financial constraints and the COVID-19 pandemic (Figure 1).

Sampling method

Simple random sampling was done from patients who underwent endoscopy of the upper gastrointestinal tract (UGIT) and tested positive for RUT. We used simple random sampling methods to ensure an unbiased representation of patients who undergo endoscopy of the upper UGIT and test positive for the RUT.

Data collection and intervention

Questionnaire

Information on clinical history and adverse effects during triple therapy was collected using a questionnaire administered by the investigator.

Intervention

Patients meeting the inclusion criteria were randomly assigned into two different therapeutic intervention programs: (i) Group A (experimental group) (n = 50), which was given amoxicillin (1000 mg, two times a day), levofloxacin (500 mg once daily), rabeprazole (20 mg two times a day) with probiotics *Lactobacillus plantarum* LA 301, *Lactobacillus salivarius* LA 302 (once daily) for 14 days, and (ii) Group B (control group) (n = 50), which was given amoxicillin (1000 mg, two times a day), levofloxacin (500 mg once daily), and rabeprazole (20 mg two times a day for 14 days).

Follow-up was done six weeks after therapy completion. This follow-up time point allowed for assessing ADRs and abrogation rates of *H. pylori* following the intervention. During the follow-up, a second review endoscopy of UGIT with RUT was conducted to determine the status of *H. pylori* infection. During the follow-up, any ADRs experienced by the patients during the triple therapy were recorded.

Statistical analysis

No participants missed the follow-up after the intervention; thus, intention-to-treat analysis was introduced here. An independent sample t-test for continuous observation and a chi-square test for categorical observation were used to assess the demographic features. A multiple logistics regression model was used to estimate the risk of adverse effects in triple therapy with probiotics group compared to triple therapy without probiotics. The independent factors influencing the model >5% were used as covariates in the final regression model, i.e., age, sex, and history of hypertension, multiple comorbidities, smoking, and taking NSAIDs. Longitudinal multilevel mixed-effect models were used to assess the overall change in RUT after triple therapy with probiotics intervention. Models were adjusted with covariates (age, sex, history of morbidities, and smoking history) that affected the model R2 by 5% or more in the best-fitted regression model; additionally, time was used as a covariate to reduce the multicollinearity. For statistical analysis, Stata Statistical Software: Release 15 (StataCorp LLC, College Station, Texas, United States) and GraphPad Prism version 8.3.0 (Insightful Science, LLC, San Diego, California, United States) were used for graphical presentation. A p-value of p<0.05 was considered as significant.

## Results

The participants in Group A (probiotics group) had an average age of 37.4 years, with a standard deviation of 13.4 years, while those in Group B (non-probiotics control group) had an average age of 38.3 years, with a standard deviation of 14.1 years. Regarding gender distribution, 52% of the probiotics group were male, and 48% were female. In the non-probiotics group, 54% were male, and 46% were female (Table 1).

**Table 1:** Demographic characteristics of the study participants Data have been presented as n (%) except for age, which has been presented as mean±SD. An Independent sample t-test for continuous data and chi-square for categorical data was used to estimate the p-value. An unpaired t-test was conducted. NSAID: non-steroidal anti-inflammatory drugs

Variables	Triple therapy with probiotics (N=50)	Triple therapy without probiotics (N=50)	p-value
Age (years), mean±SD	37.4±13.4	38.3±14.1	0.744
Sex, n (%)			
Male	26 (52.0%)	27 (54.0%)	0.841
Female	24 (48.0%)	23 (46.0%)	
History of comorbidities, n (%)			
Diabetes	6 (12.0%)	7 (14.0%)	0.766
Hypertension	13 (26.0%)	7 (14.0%)	0.134
Others	4 (8.00%)	2 (4.00%)	0.400
Multiple comorbidities	1 (2.00%)	2 (4.00%)	0.558
Nil	33 (66.0%)	38 (76.0%)	0.271
Personal history, n (%)			
Smoking	11 (22.0%)	9 (18.0%)	0.617
Alcohol	2 (4.0%)	1 (2.0%)	0.558
Tea/coffee	18 (36.0%)	16 (32.0%)	0.673
Betel nut	8 (16.0%)	15 (30.0%)	0.096
Drug history, n (%)			
NSAIDs	7 (14.0%)	8 (16.0%)	0.779

The breakdown of comorbidities in both groups showed a nearly identical distribution, covering conditions such as diabetes, hypertension, others, and multiple comorbidities. The category 'Nill' indicated participants with no comorbidities. Among the participants in the probiotics group, 22% were smokers, while in the non-probiotics group, 18% were smokers. Additionally, 36% of the probiotics group reported consuming tea or coffee, compared to 32% in the non-probiotics group. Regarding betel nut consumption, 30% of participants in the non-probiotics group reported its intake, whereas only 16% of participants from the probiotics group had a history of betel nut consumption. The history of NSAIDs showed a similar distribution in both groups (Table 1).

In the group receiving triple therapy with probiotics (N=50), 50% experienced abdominal pain, 16% had anorexia, 34% reported nausea, 8% had episodes of vomiting, 56% suffered from dyspepsia, and 56% had constipation. In the group receiving triple therapy without probiotics (N=50), 48% experienced abdominal pain, 22% had anorexia, 20% reported nausea, 4% had episodes of vomiting, 42% suffered from dyspepsia, and 44% had constipation (Table 2).

**Table 2:** History of present illness of the study subjects Data have been presented as n (%). A chi-square test was conducted.

	Triple therapy with probiotics (N=50), n (%)	Triple therapy without probiotics (N=50), n (%)	p-value
Abdominal pain	25 (50.0)	24 (48.0)	0.841
Anorexia	8 (16.0)	11 (22.0)	0.444
Nausea	17 (34.0)	10 (20.0)	0.115
Vomiting	4 (8.0)	2 (4.0)	0.400
Dyspepsia	28 (56.0)	21 (42.0)	0.161
Constipation	27 (56.0)	22 (44.0)	0.317

The history of past illnesses, including hematemesis and melena, exhibited no significant difference between the two groups (p=1.000). Personal habits such as smoking, alcohol consumption, tea/coffee consumption, betel nut use, and consumption of spicy and oily food also did not significantly differ between the two groups (p>0.05). The family history of PUD was reported by 32% of subjects in the probiotics group and 28% in the non-probiotics group, with no significant difference (p=0.663). The history of taking NSAIDs was also similar between the two groups (p=0.779) (Table 3).

**Table 3:** History of past illness, personal history, family history of PUD, and history of drugs Data have been presented as n (%). A chi-square test was conducted. PUD: peptic ulcer disease

	Triple therapy with probiotics (N=50), n (%)	Triple therapy without probiotics (N=50), n (%)	p-value
History of past Illness			
Hematemesis	0 (0.0)	1 (2.0)	1.000
Melena	4 (8.0)	5 (10.0)	1.000
Personal history			
Smoking	11 (22.0)	9 (18.0)	0.617
Alcohol	2 (4.0)	1 (2.0)	1.000
Tea/coffee	18 (36.0)	16 (32.0)	0.673
Betel nut	8 (16.0)	15 (30.0)	0.096
Spicy and oily food	18 (36.0)	19 (38.0)	0.836
Family history of PUD	16 (32.0)	14 (28.0)	0.663
History of taking NSAID	7 (14.0)	8 (16.0)	0.779

Table 4 categorizes findings into reflux esophagitis, erosive gastritis, gastric ulcer, and duodenal ulcer, providing the respective percentages within each group. The p-values, derived from the Chi-Square test, are included to assess the statistical significance of differences in endoscopic findings between the two treatment groups. However, the results indicate no statistically significant variations in the prevalence of reflux esophagitis, erosive gastritis, gastric ulcer, or duodenal ulcer between the groups, as all the p-values surpass the conventional significance threshold of 0.05.

**Table 4:** Findings of initial endoscopy The p-value was estimated using the Chi-Square test.

	Triple therapy with probiotics (N=50), n (%)	Triple therapy without probiotics (N=50), n (%)	p-value
Reflux esophagitis	25 (50.0)	24 (48.0)	0.952
Erosive gastritis	17 (34.0)	18 (36.0)	0.822
Gastric ulcer	5 (10.0)	6 (12.0)	0.911
Duodenal ulcer	3 (6.0)	2 (4.0)	0.871

When we assessed the endoscopy findings during the follow-up, it was detected that there was no significant variation between the two groups (Table 5).

**Table 5:** Findings of the review endoscopy during follow-up The P-value was estimated by using the Chi-Square test.

	Triple therapy with probiotics (N=50), n (%)	Triple therapy without probiotics (N=50), n (%)	p-value
Reflux esophagitis	10 (20.0)	11 (22.0)	0.910
Erosive gastritis	21 (42.0)	24 (48.0)	0.899
Gastric ulcer	8 (16.0)	5 (10.0)	0.523
Duodenal ulcer	2 (4.0)	2 (4.0)	0.999
Normal	9 (18.0)	8 (16.0)	0.912

**Figure 2:**
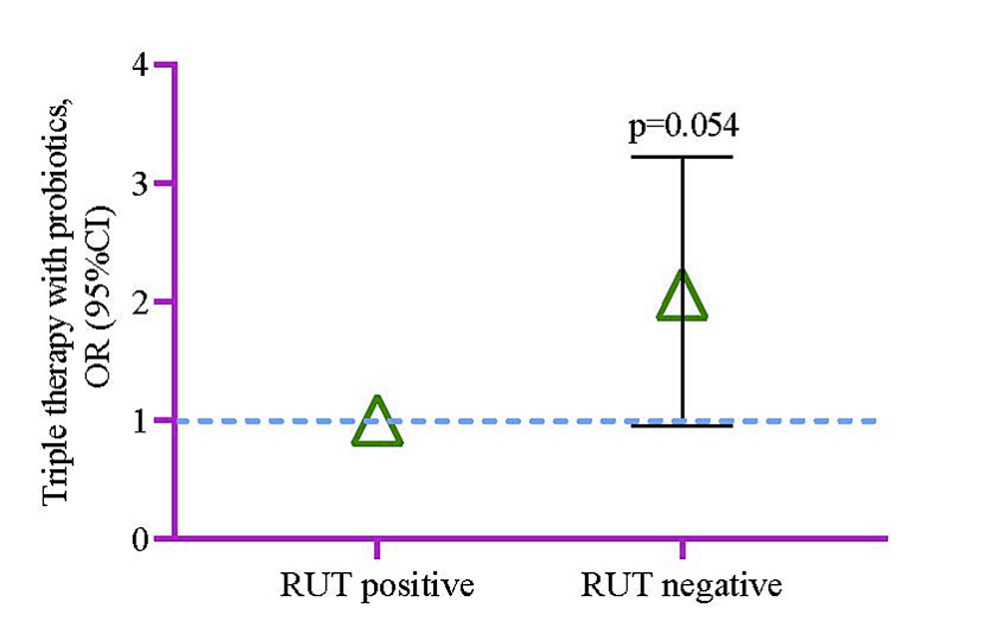
The odds ratio of RUT risk becoming negative in the supplementation group compared to the group without probiotics. A multilevel mixed-effect model was used to analyze the OR of RUT. The analysis considered potential confounders that affected the model by >5% (age, sex, history of morbidities, and smoking history). RUT: rapid urease test Image Credit: Md. Ahsanul Haq

Longitudinal multilevel was used to assess the overall change in RUT after triple therapy with probiotics intervention. The odds of RUT becoming negative from positive were 2.06 times higher (95%CI 0.95-3.22, p=0.054) in the probiotics group compared to the non-probiotics control group (Figure 2). When the association was checked with a non-parametric approach, ADRs were significantly higher in the non-probiotics group (p=0.045) than in the probiotics group (Table 6).

**Table 6:** Adverse Effects of Triple Therapy on the Study Subjects (N=100) Notes: A Chi-Square Test was Conducted.

Adverse Drug Reactions of Triple Therapy	Triple Therapy with Probiotics (n=50)	Triple Therapy without Probiotics (n=50)	p-value
Yes	19 (38.0)	29 (58.0)	0.045
No	31 (62.0)	21 (42.0)	

**Figure 3:**
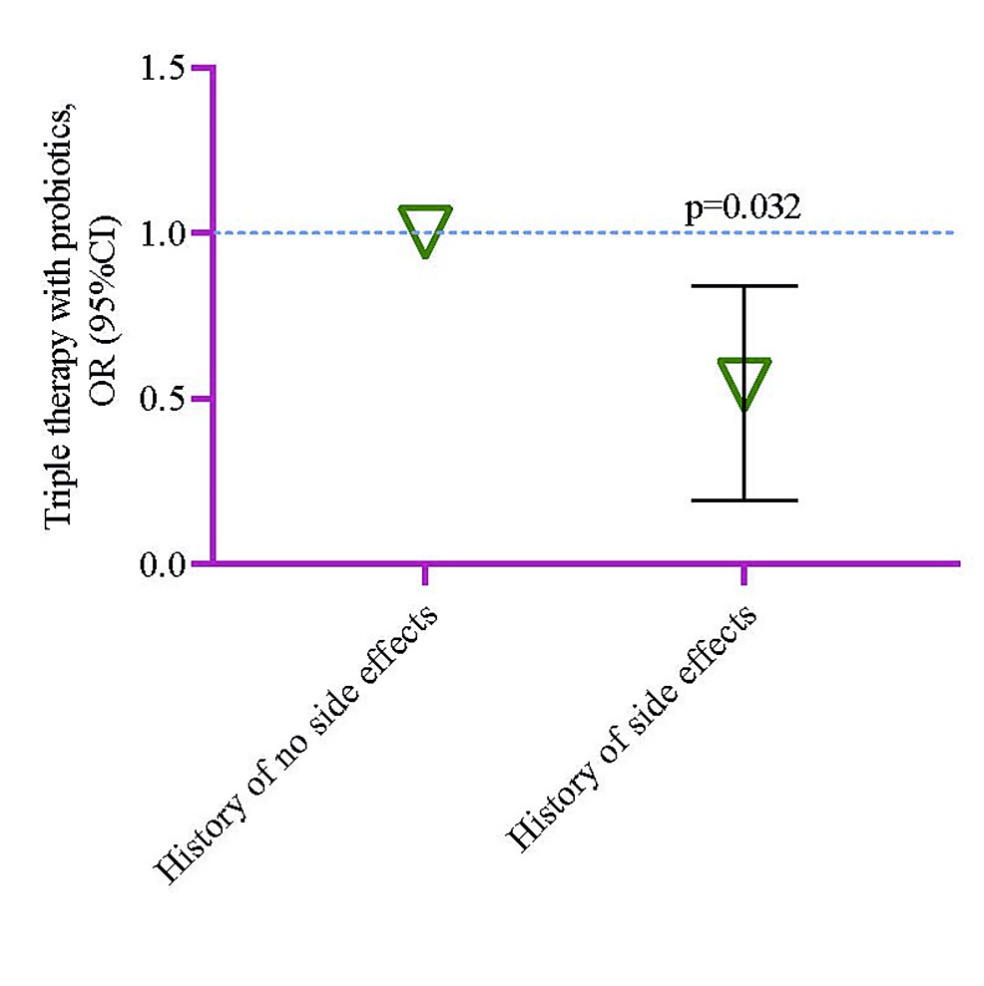
Risk of having adverse effects of triple therapy in the probiotics group compared to the non-probiotic group. The logistic regression model was used to estimate the p-value, and the regression model was adjusted by age, sex, history of hypertension, history of multiple comorbidities, history of smoking, and history of taking NSAIDs. NSAID: non-steroidal anti-inflammatory drugs Image Credit: Md. Ahsanul Haque.

When the history of ADRs in the probiotics group was compared to the non-probiotics control group by the logistic regression model, it was observed that the probiotics group had a lower risk of having detrimental effects by 0.54 times (95%CI 0.19-0.84, p=0.032) (Figure 3). 

**Figure 4:**
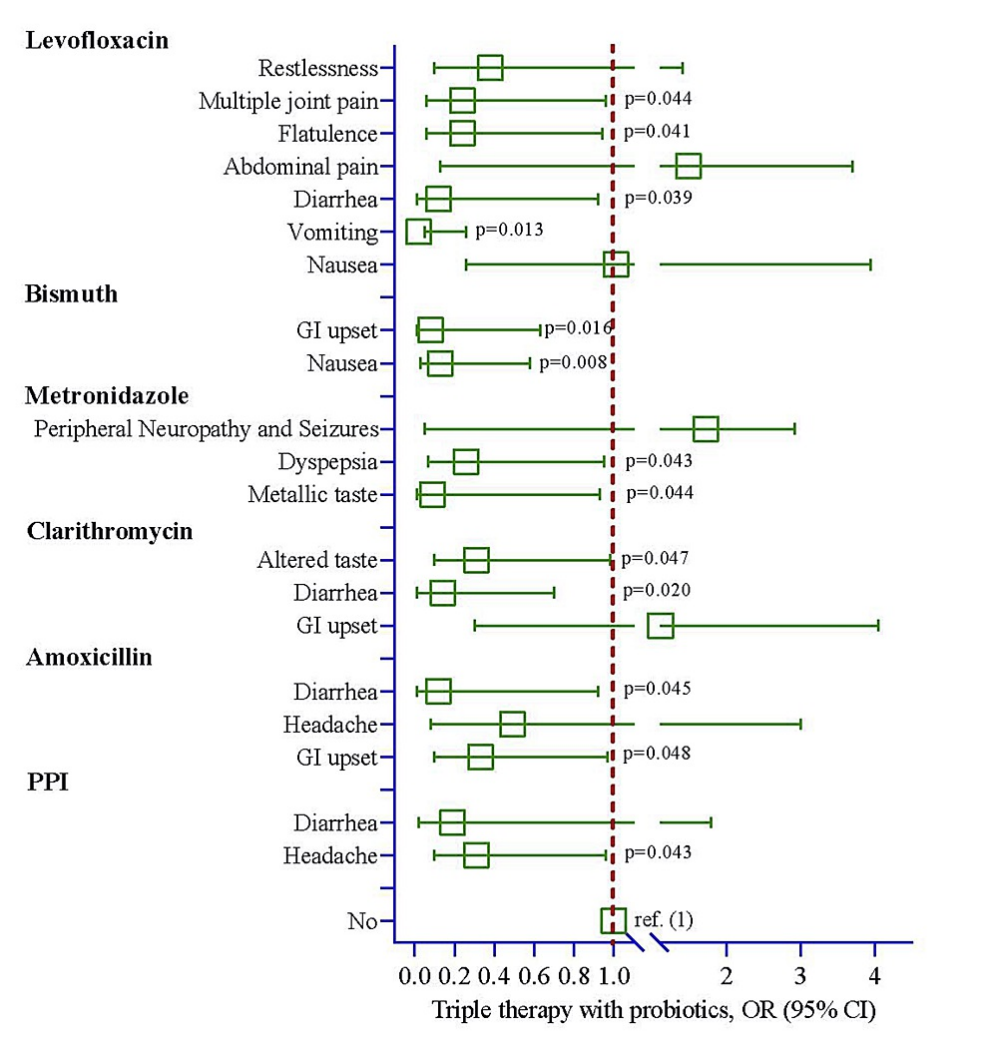
Risk of adverse effects in triple therapy with probiotics group compared to triple medications excluding probiotics. No adverse impact was used as reference (1) for all measured events. The logistic regression model was used to estimate the p-value, and the regression model was adjusted by age, sex, history of hypertension, history of multiple comorbidities, history of smoking, and history of taking NSAIDs. NSAID: non-steroidal anti-inflammatory drug; PPI: proton pump inhibitor Image Credit: Md. Ahsanul Haq

Upon stratifying the risk of ADRs based on antibiotic usage and employing a logistic regression model, the analysis revealed significantly lower odds of experiencing headaches in the group using PPI, antibiotics with probiotics, with an OR of 0.31 (95%CI 0.10-0.96, p=0.043) as depicted in Table 7 and Figure 4. Furthermore, in the amoxicillin group, the likelihood of GI upset and diarrhea was reduced by 0.33 and 0.12 times, respectively, in the presence of probiotics. For those study participants taking clarithromycin, the risk of diarrhea was 0.14 times lower in the group with probiotics compared to the group without probiotics. The analysis also indicated a 0.26 times lower risk of dyspepsia in the group with probiotics for individuals using metronidazole. Bismuth users exhibited a decreased risk of nausea and GI upset by 0.13 and 0.08 times, respectively. Moreover, probiotic users demonstrated lower odds of experiencing vomiting (OR=0.02), diarrhea (OR=0.12), flatulence (OR=0.24), and multiple joint pain (OR=0.24) when using levofloxacin as antibiotics (Table 7 and Figure 4).

**Table 7:** Risk of adverse effects of the antibiotics in triple therapy with probiotics compared to triple therapy without probiotics Multinomial logistic regression was used to estimate the p-value, and the regression model was adjusted by age (category), sex (type, male and female), history of hypertension (category), history of multiple comorbidities (categorical), smoking history (categorical), history of taking NSAID (categorical). NSAID: non-steroidal anti-inflammatory drug; PPI: proton pump inhibitor

Drug	Adverse Drug Reactions	Triple therapy without probiotics (N=50)	Triple therapy with probiotics (N=50), OR (95%CI)	p-value
PPI	Headache	1	0.31 (0.10, 0.96)	0.043
	Diarrhea	1	0.19 (0.02, 1.79)	0.145
Amoxicillin	GI upset	1	0.33 (0.10, 0.97)	0.048
	Headache	1	0.49 (0.08, 3.00)	0.437
	Diarrhea	1	0.12 (0.01, 0.92)	0.045
Clarithromycin	GI upset	1	1.11 (0.30, 4.05)	0.882
	Diarrhea	1	0.14 (0.01, 0.70)	0.020
	Altered taste	1	0.31 (0.10, 0.98)	0.047
Metronidazole	Metallic taste	1	0.09 (0.01, 0.93)	0.044
	Dyspepsia	1	0.26 (0.07, 0.95)	0.043
	Peripheral neuropathy and seizures	1	1.72 (0.05, 2.92)	0.766
Bismuth	Darkening of the tongue and stool	1	-	
	Nausea	1	0.13 (0.03, 0.58)	0.008
	GI upset	1	0.08 (0.01, 0.63)	0.016
Levofloxacin	Nausea	1	1.01 (0.26, 3.94)	0.983
	Vomiting	1	0.02 (0.05, 0.26)	0.013
	Diarrhea	1	0.12 (0.01, 0.92)	0.039
	Abdominal pain	1	1.48 (0.13, 6.69)	0.754
	Flatulence	1	0.24 (0.06, 0.94)	0.041
	Multiple joint pain	1	0.24 (0.06, 0.96)	0.044
	Restlessness	1	0.38 (0.10, 1.40)	0.145

## Discussion

**Figure 5:**
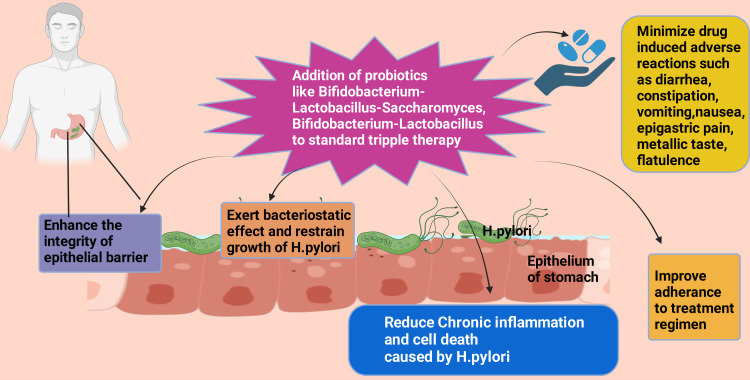
Beneficial effects of adding probiotics to the standard triple therapy on patients with H. pylori infection. *H. pylori*: *Helicobacter pylori* Image credit: Rahnuma Ahmad; with the premium version of BioRender [114] with license number CH26GB4NBY.

Table 8 gives details of selected studies from the literature regarding *H. Pylori* eradication and dysbiosis. The STT for eliminating *H. pylori* often upsets intestinal ﻿microbiota, especially the antimicrobial component of the therapy [113-117]. Additionally, it causes vitamin (vitamin A, B, C, K, α-tocopherol, vitamin B12, and folic acid) insufficiency in the human body [116-118]. Various studies revealed that *H. pylori* extinction treatment regimens have raised the possibility of several pathogenic microbes such as *Streptococcus*, *Klebsiella*, and *Shigella* [119,120]. These pathogens instigate chronic inflammation and promote the synthesis of genotoxins or carcinogenic metabolites [121-123]. This chronic inflammation of stomach-related gastric dysbiosis bed frequently causes DNA alteration and upholds precancerous ulceration [28,124-128]. *H. pylori*-related gastric precancerous situation ultimately turned to atrophic gastritis and finally carcinoma with a very high mortality rate [129,130]. Conversely, multiple studies reported that administering probiotics before and after eradication therapy for *H. pylori* rapidly reinstates intestinal microbiota (Figure 5), especially *Bacillus* and Lactic acid bacteria [117,131,132]. Another issue is antimicrobial resistance regarding *H. pylori *management that leads to pharmacological intervention failure throughout the globe [66,80,133-136].

**Table 8:** Selected randomized control trials, systematic review, review, and meta-analysis regarding H. Pylori eradication and dysbiosis Keywords were “H. Pylori,” “Eradication,” and “Dysbiosis.” Filter Applied: Randomized Control Trials, Systematic Review, Review, and Meta-Analysis. Indexed in PubMed. H. pylori: Helicobacter pylori

Type of Study	Authors, year, reference	Background	Result	Conclusion
Randomized Controlled Trials	He et al., 2022 [131]	The use of antibiotics to treat *H. pylori *in the gut causes derangement in microbiota. Probiotics reduce the adverse effects of antibiotics, but their impact on allaying microbiota is not well-established	The elimination rate of *H. Pylori* was almost similar in the placebo and probiotic groups. Normal commensals suppressed following eradication were replaced by probiotics, and microbiota was progressively restored within two weeks. Adverse events were also less in the probiotic cluster.	Concurrent use of probiotics in *H. pylori* elimination mitigates unwanted effects and restores normal commensals in the gut and oral cavity.
	Yuan et al., 2021 [137]	The effect of probiotics on gut microbiota in both *H. pylori*-positive and *H. pylori*-negative patients is not apparent.	Clusters treated with quadruple therapy and probiotics had increased levels of commensals, e.g., Bifidobacterium and lactobacillus. Probiotics alone were not successful in the elimination of *H. pylori.* Only quadruple therapy caused significant disruption of the gut microbiome.	*H. pylori* elimination causes alleviation of normal flora in GIT. This disruption can be minimized if probiotics are added to the treatment protocol.
	Chen et al., 2021 [138]	Disruption of gut commensals during H pylori. The use of probiotics can alleviate H.pylori treatment. However, data on the role of probiotics alone in suppressing Helicobacter infection is scarce.	*H. pylori* load was reduced with Lactobacillus acidophilus and L. rhamnosus, but the gut microbiome did not exhibit any significant variation.	Specific probiotics may mitigate* H. pylori* load without altering the gut microbiota.
	Dore et al., 2022 [139]	Treatment of *H. pylori* is always accompanied by the development of adverse effects, primarily on the gut microbiome. The use of probiotics can reduce this side effect.	There was no significant difference in the eradication rate of *H. pylor*i. Gut microbiota was similarly deranged by *L. reuteri *and bismuth and restored after 30-40 days.	*L.reuteri *can be used in patients who cannot tolerate bismuth along with low-dose quadruple therapy, but disturbance of gut microbiome was unavoidable
	Guillemard et al., 2021 [140]	Alteration of gut microbiota during *H. pylori* treatment is now well established. This derangement can be mitigated using probiotics, but it is poorly documented. A fermented milk product containing several strains was assessed in this research.	The test group did not have a significant impact on GIT symptoms. Still, beta diversity was less, the number of pathogenic bacteria was reduced, and that of short-chain fatty acid (SCFA) was increased.	This seven-strain fermented milk can be helpful to ensure brisk retrieval of the gut microbiome after* H. pylori* treatment.
	Yakovenko et al., 2021 [141]	*H. pylori* removal by quadruple therapy containing bismuth also causes various unwanted GI symptoms. The addition of probiotics may alleviate these adverse events	When a Bifiform capsule was added to the regimen, disruption of colonic microbiota was less, plasma cells were increased, and IgA level was stable.	Incorporation of probiotics in the treatment regimen can reduce side effects and increase the immunity of the gut.
Systematic Review and Meta-analysis	Guo et al., 2022 [142]	Alteration of gut microbiome is noticed during *H. Pylor*i elimination. Whether gut microbiota is restored in its pre-infection state after treatment is not documented.	Successful elimination of *H. pylori* was noticed in both quadruple and triple therapy. Alpha diversity and alpha and beta diversity were documented in quadruple and triple treatment, respectively.	*H. pylori*, the available regimen can successfully eliminate pylori, but a conclusion on restoration of gut commensals could not be drawn.
Review	Suzuki et al., 2022 [143]	*H. pylori *is responsible for gastric inflammation, peptic ulcer, and stomach cancer. Treatment protocols show diversity in different provinces of the world.	Based on present evidence, quadruple without bismuth and vonoprazan-based triple therapy was 90% effective but still caused a disturbance in gut microbiota.	A less costly, more straightforward regimen that will not cause the risk of dysbiosis is needed. Dual therapy with amoxicillin and vonoprazan may be considered.

Tables 9-10 give the list of studies reviewed on *H. Pylori *resistance in Bangladesh and low- and middle-income countries, respectively.

**Table 9:** Selected studies indexed in PubMed on Helicobacter pylori resistance in Bangladesh Keywords were “Helicobacter Pylori,” Resistance,” and “Bangladesh.”

Study (Authors, year, reference)	Background	Result	Conclusion
Fauzia et al., 2023 [144]	Due to the development of resistance to usual antibiotics, treatment against *H. pylori *has become less effective. Clinical detection is formidable. So, understanding the fundamental mechanism is critical to fight against resistance.	Resistance was highest against clarithromycin, followed by levofloxacin, amoxicillin, and metronidazole. Multi-drug resistance was observed in most of the cases.	These findings may facilitate fast decisions on the susceptibility of antibiotics against *H. pylori*.
Aftab et al., 2016 [74]	Knowledge of antibiotic resistance during H.pylori treatment is crucial, but the unavailability of current data makes treatment arduous.	Metronidazole and clarithromycin were the topmost resistant antibiotics, and levofloxacin is rising. Lower resistance was documented in the case of tetracycline and amoxicillin.	In Bangladesh, resistance against metronidazole, clarithromycin, and levofloxacine were highest, making the triple therapy futile.
Shrestha et al., 2023 [145]	Antimicrobial resistance against *H. pylori* has become common in South Asia, but data is limited.	The top five antibiotics found to be resistant were Metronidazole, levofloxacin, clarithromycin, amoxicillin, and tetracycline. India, Pakistan, and Bangladesh were the most prevalent countries regarding resistance.	Antibiotic resistance is on the rise among South Asian countries, and most include commonly used drugs.
Miftahussurur et al., 2019 [146]	Antibiotic resistance against *H. pylori* is highly prevalent in Bangladesh and Nepal. Finding an alternative protocol is now a time-demanding issue. For that, knowledge of the resistance rate and its mechanism is crucial.	Rifabutin, furazolidone, and sitafloxacin were highly susceptible, and rifaximin was resistant in both countries. Samples from Bangladesh showed higher resistance against garenoxacin. Mutations in A87, D91, and gyrA were responsible for developing resistance.	Furazolidone and sitafloxacin can be added to the treatment regimen in resistant cases. Rifabutin should be used.
Qaria et al., 2018 [147]	*H. pylori* cell wall contains Cholesteryl glucosides (CGs), which makes the bacteria pathogenic and virulent. Deleting the gene responsible for this enzyme formation may cause the bacteria to be vulnerable to available antibiotics.	Morphological alterations were perceived following the deletion of the responsible gene, making the bacteria more vulnerable to commonly used antibiotics.	The absence of CGs converts resistant *H. pylori *to a susceptible one due to cell wall modification.
Fauzia et al. , 2023 [148]	*H. pylori* is a highly biofilm-forming organism that ensures protection against antibiotics. Evaluation of the underlying mechanism of biofilm formation is, therefore, very crucial	Most of the strains were found to be low biofilm forms. G160S, N156K, and A223V mutations among the nucleotide polymorphisms (SNP) were responsible for high biofilm formation in Bangladeshi people.	SNPs have a vital role in biofilm construction, and the method applied in the research can be used to detect mutations.
Reza et al., 2023 [149]	Effective treatment of *H. Pylori* infection is vital as it may result in gastric carcinoma. Due to the development of resistance, antimicrobials have become less potent. Gene therapy can be an efficient alternative.	Specifically designed siRNA were successful in silencing the sequence-specific gene.	These designed siRNAs can be incorporated in treating *H. pylori* causing gastric carcinoma.
Banatvala et al., 1994 [150]	Antibiotic resistance, especially of metronidazole, is well reported in people of developing countries, but data is scarce on the transfer of these resistant varieties through migrants.	The resistant variant was higher in migrants, with the majority from Bangladesh. Women born in the United Kingdom and previous treatment with nitroimidazole, metronidazole, and tinidazole were other risk factors.	Migrants affected by *H. pylori* from developing countries will likely have resistant varieties.
Khan et al., 2004 [151]	Treatment against *H. pylori* often fails due to resistance against clarithromycin. Alteration in genomic sequence is responsible for developing resistance, but data is limited in cases from Bangladesh.	A T-to-C transition mutation at position 2182 was found in every clarithromycin-resistant case that was analyzed.	Resistance against clarithromycin develops due to a T-to-C transition mutation at position 2182 of the *H. pylori *genome.
Nahar et al., 2004 [73]	Eliminating *H. pylori* often fails due to antimicrobial resistance against commonly used antibiotics, but data is scarce in Bangladesh.	Metronidazole, followed by tetracycline, clarithromycin, and amoxicillin, were primarily resistant.	Higher resistance against common antibiotics in Bangladesh proves the need for extended monitoring.
Qumar et al., 2021 [152]	*H. pylori* infection is mostly prevalent in developing countries, and its treatment has become arduous due to the emergence of resistant varieties. The genotype of the predominant variety is not well documented in Bangladesh.	Isolated strains showed two separate varieties, e.g., HpAsia2 and HpEurope, where the prior one was more virulent.	The genotype of the bacteria, as well as environmental and host factors, are crucial to determine the clinical prognosis.
Khan et al. , 2008 [153]	Mutations in the 16S rRNA gene are thought to be responsible for clarithromycin-resistant *H. pylori* infection cases. Data from Bangladesh were limited.	In the tetracycline-resistant variety, none showed a mutation in the 16S rRNA gene.	Tetracycline resistance may also appear due to other causes rather than RNA mutation.
Rahman et al., 2009 [154]	Cytotoxin-associated gene A (CagA) of *H. pylori *is a risk factor for developing coronary heart disease (CHD). Data on this association among Asian Indians are sparse.	*H. pylori*-positive cases with or without CHD had lower levels of HDL, while fasting plasma glucose was markedly increased in HP+ve patients with CHD. Insulin secretory dysfunction (ISD) was higher in all infected cases compared to control groups.	*H. pylori* infection is a risk factor for developing CHD and Diabetes mellitus.
Akash et al. , 2023 [155]	Inflammation of gastric mucosa by *H. pylori* may lead to the development of gastric cancer. The rising tendency of antimicrobial resistance has further increased the risk, prompting the need for alternative treatment.	Among the eight phytocompounds, Sarsasapogenin and Diosgenin showed the highest binding affinity, and the rest showed superiority over mitomycin.	Gastric carcinoma, induced by *H. pylori*, may respond to these natural compounds, but further studies are required.
Rimbara et al., 2007 [156]	Nine genomic mutations were thought to be responsible for the development of clarithromycin resistance of *H. pylori*, but documents from Japan are not well-concluded	An adenine → guanine transition at position 2143 (A2143G) or 2142 (A2142G) was detected in every clarithromycin-resistant case.	In Japan, mutations in positions 2142 and 2143 were associated with resistance to clarithromycin.

**Table 10:** Selected consequential papers on Helicobacter pylori resistance in low and middle-income countries. Keywords were “Helicobacter Pylori,” “Resistance,” and “Low and middle-income countries.” Filters Applied: Free full text, Meta-Analysis, Randomized Controlled Trial, Review, Systematic Review in the last five years. Indexed in PubMed.

Type of Study	Authors, year, reference	Background	Result	Conclusion
Review	Qiu et al., 2021 [157]	Noninvasive investigations, e.g., urea breath test, are preferable for detecting *H. pylori*. Due to the high cost, lack of expertise, and non-compliance of child patients, detecting bacteria from stool samples may be a suitable alternative.	Commercially available kits for stool antigen tests (SAT) showed promising results for mass screening. Immunoassay also demonstrated higher specificity and sensitivity. PCR is also advantageous but costly.	Identifying* H. pylori *from stool samples can be considered an efficient method, although it is not free of disadvantages.
Review	Nguyen et al., 2023 [158]	Children infected by *H. pylori* may remain symptom-free for a longer period but are at risk for the development of various gastrointestinal disorders later. Comprehensive data are lacking on childhood infection.	The prevalence of *H. pylori* is still high in the pediatric age group. Lack of symptoms makes diagnosis difficult, followed by the development of antibiotic resistance.	PCR-based diagnosis and detection of susceptibility are essential for successful eradication. During prevention, host and environmental factors should be taken into consideration.
Review	Mestre et al., 2022 [97]	Incorporating probiotics with standard treatment protocol for eradicating *H. pylori* exerts better results and mitigates side effects.	Probiotics alone contribute to the restoration of the gut microbiome only, but when added to the treatment regimen, the treatment success rate is higher, and adverse events are less.	Data from the Asian population has revealed the efficacy of probiotics, but more studies are needed from other parts of the world.
Review	Garrido-Treviño et al., 2022 [159]	Treatment of *H. pylori* infection is often given empirically, which has played a vital role in developing resistant strains. Noninvasive tests such as molecular methods may alleviate this disadvantage.	Higher sensitivity and specificity were observed in molecular methods in detecting *H. pylori*.	New-generation molecular methods can help mitigate antimicrobial resistance.
Systematic review and meta-analysis	Wang et al., 2023 [160]	Antibiotic-resistant varieties constantly disrupt the elimination of *H. pylori*. Data on primary antibiotic resistance is not well documented in China.	Resistance was found to be higher in the case of metronidazole, clarithromycin, and levofloxacin, followed by amoxicillin.	Commonly used antibiotics showing resistance may result in treatment failure and require rapid action.
Systematic Review	Rojas et al., 2021 [161]	There are several diagnostic methods for *H. pylori* infection, but they differ in cost and effectiveness.	Rapid, noninvasive methods are now replacing invasive tests. Detection of antibiotic resistance by various methods is also being included at a higher rate, but studies on the effectiveness of different techniques are still limited.	Using diagnostic methods instead of empirical therapy may lessen the economic burden.
Systematic Review	Sukri et al., 2021 [162]	Antimicrobial resistance, especially against clarithromycin, is a significant obstacle in eradicating *H. pylori*. Alternative treatment is now needed, especially for people with limited resources.	Data from different Southeast Asian countries showed higher resistance to metronidazole, clarithromycin, and levofloxacin. Antimicrobial peptides (AMP) were found to be effective in resistant cases.	AMPs can be incorporated into treatment protocol to combat resistant variety.
Randomized Control Trial	Vilaichone et al., 2020 [163]	In Bhutan, gastric cancer induced by* H. pylori* infection is the commonest among malignancies. Therefore, a cost-effective method for *H. pylori* elimination is critical in reducing this malignancy.	Fourteen-day regimens showed a higher eradication rate; females and patients over 40 were more responsive.	A 14-day regimen can be more effective in economically constrained areas like Bhutan.
Meta-Analysis	Fontes et al., 2019 [164]	As antibiotic resistance against *H. pylori* is on the rise, alternative therapy such as N-acetylcysteine (NAC) is now very important, especially for regions with limited sources.	The quality of methodology was poor in the included studies. However, NAC did not show significant efficacy over conventional treatment.	A definitive conclusion could not be drawn on the efficacy of NAG regarding *H. pylori *treatment.

**Figure 6:**
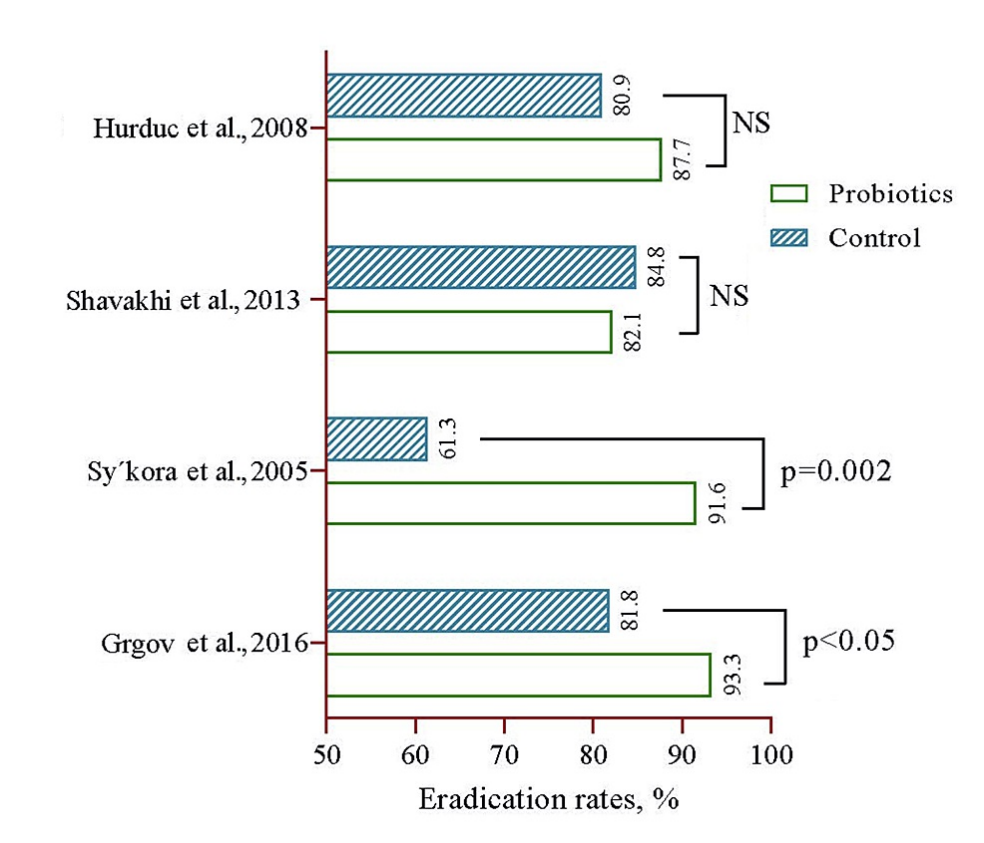
Selected consequential papers showing the benefits of adding probiotics with standard therapy for Helicobacter pylori eradication. Notes: Keywords were “Benefits,” “Probiotics,” “*Helicobacter pylori*,” “Eradication,” and “Triple therapy.” Filters Applied: Randomized Controlled Trials. Indexed in PubMed. References: [165-168] Image Credit: Md. Ahsanul Haq

Additionally, Grgov et al. [165] and Sýkora et al. [166], in their randomized control trials, revealed that probiotic treatment led to significantly higher eradication rates of 93.3% and 91.6%, respectively, compared to the control groups. On the contrary, Shavakhi et al. [167] and Hurduc et al. [168] observed no significant difference between the two treatment groups (Figure 6).

One earlier study reported that usual ADRs observed in the combined triple therapy + probiotics group were dyspepsia (4.4%), nausea and/or vomiting (2.9%), dry mouth (2.6%), diarrhea (1.5%), and abdominal pain (1.1%) [169]. However, the current study uncovered that among patients who received triple therapy with probiotics, common ADRs included abdominal pain, anorexia, nausea, vomiting, and dyspepsia. So, our study's findings showed that the types of ADRs and the incidence rate were similar. Furthermore, patients receiving triple therapy without probiotics exhibited higher levels of common similar ADRs (Table 6). Akcam et al. reported identical observations [170]. Furthermore, Lü et al. reported in their meta-analysis comprising 13 randomized controlled trials that adding probiotics throughout *H. pylori* eradication therapy reduces ADRs [171]. 

No significant differences were observed in the initial and follow-up endoscopic findings regarding reflux esophagitis, erosive gastritis, gastric ulcer, and duodenal ulcer between the experimental (STT + probiotics) and control (only STT) group in the current study (Table 4) and (Table 5). One earlier study reported that *H pylori *extermination remedial measures escalate the possibility of reflux esophagitis, which is unrelated to previous records of esophagitis. Additionally, no considerable consequence can be comprehended regarding reflux-associated issues [172]. One Japanese study reported that nearly 10% of *H. pylori*-positive cases develop reflux esophagitis after pharmacological intervention and successful eradication. This observation was principally noticed among cases with severe reflux-related symptoms before medical intervention. Meanwhile, after efficacious elimination, patients with *H. pylori* had definite improvement regarding peptic-ulcer-related manifestation. Nonetheless, the possibility of the reflux esophagitis evolvement remains [173].

Model analysis revealed positive clinical outcomes concerning *H. pylori* biomarker (RUT) after triple therapy with probiotics intervention. The odds of RUT becoming negative from positive were more than two-folds higher among the experimental group (SST + probiotics) in comparison to the control group (only STT) (Figure 2). One meta-analysis by Lu et al. comprising 21 RCTs reported that additional probiotics with STT do not show clinically better outcomes regarding *H. pylori*'s extinction frequency when equated to the inactive medicinal agents [174]. Akcam et al. reported similarly that no substantial confirmation was obtained about the abolition of *H. pylori* and the minimization of ADRs [170]. Additionally, two more RCTs revealed no statistically significant differences observed between experimental (STT + probiotics) and control (only STT) [167,168].

In contrast, multiple studies reported that adding probiotics with standard therapy improves the degree of *H. pylori* eradication and diminishes antimicrobial-induced ADRs, especially gastrointestinal issues [170,175,176]. Two more RCTs revealed that adding probiotic treatment led to considerably higher eradication rates [165,166]. However, when the association was checked with a non-parametric approach, ADRs were significantly higher in the non-probiotics group than in the probiotics group (Table 6). Multiple earlier research published papers reported that the addition of probiotics with standard therapy improves the *H. pylori *eradication rate and diminishes ADRs [97,177]. One meta-analysis comprising 13 RCTs and 2306 patients revealed that adding probiotics with *H. pylori *antagonist’s regimen improves extinction percentages, parallelly lowers ADRs, and relieves most PUD-allied clinical indicators [171]. Wang et al. reported that traditional triple therapy with probiotics, especially *Bifidobacterium-Lactobacillus* and *Bifidobacterium-Lactobacillus-Saccharomyces* therapy recuperates abolition proportions and reduces ADRs [96]. Another RCT by Hauser et al. revealed that supplementation of probiotics with conventional triple therapy improves pharmacodynamic properties and decreases ADRs and patient compliance equally noticeably [178].

The logistic regression model analysis observed that the experimental group (probiotics) had a lesser risk of adverse effects when compared to the control group (without probiotics) regarding the risk of a history of ADRs in traditional triple therapy (Figure 3). Multiple studies reported that socio-anthropological differences between individual cases, history of PUD and non-communicable chronic disorders, tobacco and alcohol consumption, and the existence of hereditary issues affect extermination *H. pylori* treatment outcomes [95,179,180]. Gebeyehu et al. reported that ADRs while receiving conventional triple therapy were dependent on the following changeable features: history of PUD over 21 days, location of dwelling, body mass index (BMI), consumption of alcohol, and long intervals between meals causing abdominal pain, when conducting bivariate and multiple logistic regression analysis [88].

The logistic regression model analysis revealed significantly lower odds of experiencing headaches in the group using PPI antibiotics with probiotics (Table 7 and Figure 4). Although the exact mechanism remains obscure, adding probiotics reduces migraine-associated headaches [181]. Li et al. reported that supplementing probiotics with traditional triple therapy reduces headaches with or without vomiting [182]. Additionally, both amoxicillin and clarithromycin antimicrobials receivers the possibility of GI issues, especially diarrhea, which were reduced with the supplementation of probiotics. *Lactobacillus* strains are well-known as noble probiotics and are most widely used among humans [183-185]. Another study claimed probiotics benefit pharmacology in diverse intestinal disorders, including diarrhea [182].

Additionally, probiotics, especially *Lactobacillus* strains such as *﻿L*. *acidophilus*, *L. casei*, *L. salivarius*, *L. reuteri*, *L. johnsonii*, and *L. gasseri* [186], possess the necessary pharmacodynamics to inhibit *H. pylori* [97,186-188]. This analysis also detected a lower risk of dyspepsia in the group with probiotics for those cases receiving metronidazole. Muresan et al. reported that *Lactobacillus* strains, mainly *L. reuteri*, minimize chronic dyspepsia [188]. Bismuth salt receivers of the current study exhibited a decreased risk of nausea and GI-related disorders. Bismuth subsalicylate (BSS) was permitted by the United States Food and Drug Administration (FDA) in 1939 for following clinical disorders such as diarrhea, heartburn, indigestions, nausea, and stomach upsets [189]. Furthermore, BSS possesses pharmacological properties in minimizing GI distress and traveler's diarrhea [190]. BSS also decreases the austerity and frequency of diarrhea and flatulence [189]. Moreover, probiotic + levofloxacin recipients of the current study demonstrated lower odds of experiencing vomiting, flatulence, diarrhea, and multiple joint pain (Table 7 and Figure 4). ﻿In their research, Pugh et al. confirmed that ﻿probiotic therapeutic intervention frequently abolishes GI indicators, especially bringing up wind, ﻿biliousness, vomiting, passing intestinal gas, and loose motion [191]. Furthermore, Sheffield et al. reported that adding probiotics with fluoroquinolones does not increase ADRs [192].

**Figure 7:**
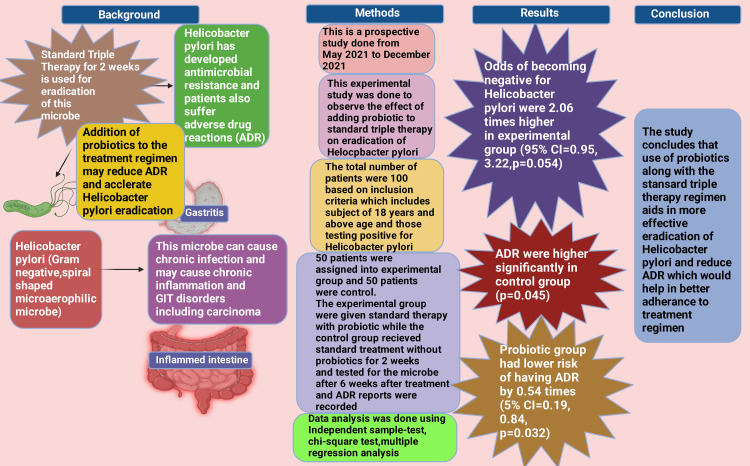
Principal findings of the current study ADR: adverse drug reaction Image Credit: Rahnuma Ahmad; with the premium version of BioRender [114] with license number XA26G8WP6M

The principal findings of the current study are illustrated in Figure 7.

Limitations of the study

This study has limitations, including a small sample size, potentially compromising the generalizability of findings. Reliance on self-reported data raises the possibility of recall bias, impacting the accuracy of information. Additional limitations include the short follow-up duration and lack of assessment for publication bias. Careful consideration of these factors is crucial for a nuanced interpretation of the study's outcomes.

## Conclusions

*H. pylori *causes persistent inflammation as well as infection in the stomach's superficial epithelial layer, leading to GI diseases such as cancer. Probiotics significantly enhance the condition of the gut microbiota and the bacteriostatic activity of probiotics hinders the replication of *H. pylori* in the stomach. Positive clinical effects for the *H. pylori *biomarker (RUT) during triple therapy with the probiotic intervention were found by model analysis in the current study. In contrast with the control group (only STT), the probability of going from positive to negative RUT was more than two times higher in the experimental group (SST + probiotics). In terms of the risk of ADRs, the logistic regression model analysis found that the experimental group (STT + probiotics) had a lower risk of side effects than the control group (only STT). Ongoing exploration is an urgent requirement among Bangladeshi patients, as *H. pylori* is highly infectious, easily transferable from person to person, and life-threatening microbes that may ultimately lead to gastric cancer. 
